# Spatio-temporal heterogeneity of malaria vectors in northern Zambia: implications for vector control

**DOI:** 10.1186/s13071-016-1786-9

**Published:** 2016-09-21

**Authors:** Jennifer C. Stevenson, Jessie Pinchoff, Mbanga Muleba, James Lupiya, Hunter Chilusu, Ian Mwelwa, David Mbewe, Limonty Simubali, Christine M. Jones, Mike Chaponda, Maureen Coetzee, Modest Mulenga, Julia C. Pringle, Tim Shields, Frank C. Curriero, Douglas E. Norris, for the Southern Africa International Centers of Excellence in Malaria Research

**Affiliations:** 1 The W. Harry Feinstone Department of Molecular Microbiology and Immunology, The Johns Hopkins Malaria Research Institute, Johns Hopkins Bloomberg School of Public Health, 615 North Wolfe Street, Baltimore, MD 21205 USA; 2 Macha Research Trust, P.O. Box 630166, Choma, Zambia; 3 The W. Harry Feinstone Department of Epidemiology, Johns Hopkins Bloomberg School of Public Health, 615 North Wolfe Street, Baltimore, MD 21205 USA; 4 Tropical Diseases Research Centre Room 727, Ndola Central Hospital, P.O. Box 71769, Ndola, Zambia; 5 Wits Research Institute for Malaria, School of Pathology, Faculty of Health Sciences, University of the Witwatersrand, Johannesburg, South Africa

**Keywords:** Malaria, Zambia, *Anopheles funestus*, *Anopheles gambiae*, Vector control

## Abstract

**Background:**

Despite large reductions in malaria burden across Zambia, some regions continue to experience extremely high malaria transmission. In Nchelenge District, Luapula Province, northern Zambia, almost half the human population carries parasites. Intervention coverage has increased substantially over the past decade, but comprehensive district-wide entomological studies to guide delivery of vector control measures are lacking. This study describes the bionomics and spatio-temporal patterns of malaria vectors in Nchelenge over a two and a half year period, investigates what household factors are associated with high vector densities and determines why vector control may not have been effective in the past to better guide future control efforts.

**Methods:**

Between April 2012 and September 2014, twenty-seven households from across Nchelenge District were randomly selected for monthly light trap collections of mosquitoes. Anopheline mosquitoes were identified morphologically and molecularly to species. Foraging rates were estimated and sporozoite rates were determined by circumsporozoite ELISAs to calculate annual entomological inoculation rates. Blood feeding rates and host preference were determined by PCR. Zero-inflated negative binomial models measured environmental and household factors associated with mosquito abundance at study households such as season, proximity to the lake, and use of vector control measures.

**Results:**

The dominant species in Nchelenge was *An. funestus* (*s.s.*) with *An. gambiae* (*s.s.*) as a secondary vector. Both vectors were found together in large numbers across the district and the combined EIRs of the two vectors exceeded 80 infectious bites per person per annum. *An. funestus* household densities increased in the dry season whilst *An. gambiae* surged during the rains*.* Presence of insecticide treated nets (ITNs) and closed eaves in the houses were found to be associated with fewer numbers of *An. gambiae* but not *An. funestus.* There was no association with indoor residual spraying (IRS).

**Conclusion:**

In Nchelenge, the co-existence of two highly anthropophagic vectors, present throughout the year, is likely to be driving the high malaria transmission evident in the district. The vectors here have been shown to be highly resistant to pyrethroids used for IRS during the study. Vector control interventions in this area would have to be multifaceted and district-wide for effective control of malaria.

**Electronic supplementary material:**

The online version of this article (doi:10.1186/s13071-016-1786-9) contains supplementary material, which is available to authorized users.

## Background

Zambia has reported considerable reductions in malaria prevalence and child mortality in the past decade. Cases of malaria in children under 5 years of age halved between 2006 and 2008 and all-cause child mortality reduced by a third between 2001 and 2008 [1]. Success has been attributed to increased coverage of vector control, mainly using long-lasting insecticide-treated nets (LLINs), improved case management using rapid diagnostic tests (RDTs) and artemether-lumefantrine, and increased uptake of intermittent preventative treatment in pregnancy (IPTp) nationally [2]. This has led to efforts to create malaria-free zones in some parts of the country. However, these gains have not been seen in all regions. An upsurge in cases was noted between 2009 and 2010 in eastern and northern Zambia [3, 4], associated with disruption of vector control [5]. In the past three years marked increases in prevalence of malaria have been seen in the north-western, Copperbelt and northern parts of the country [6].

**Fig. 1:**
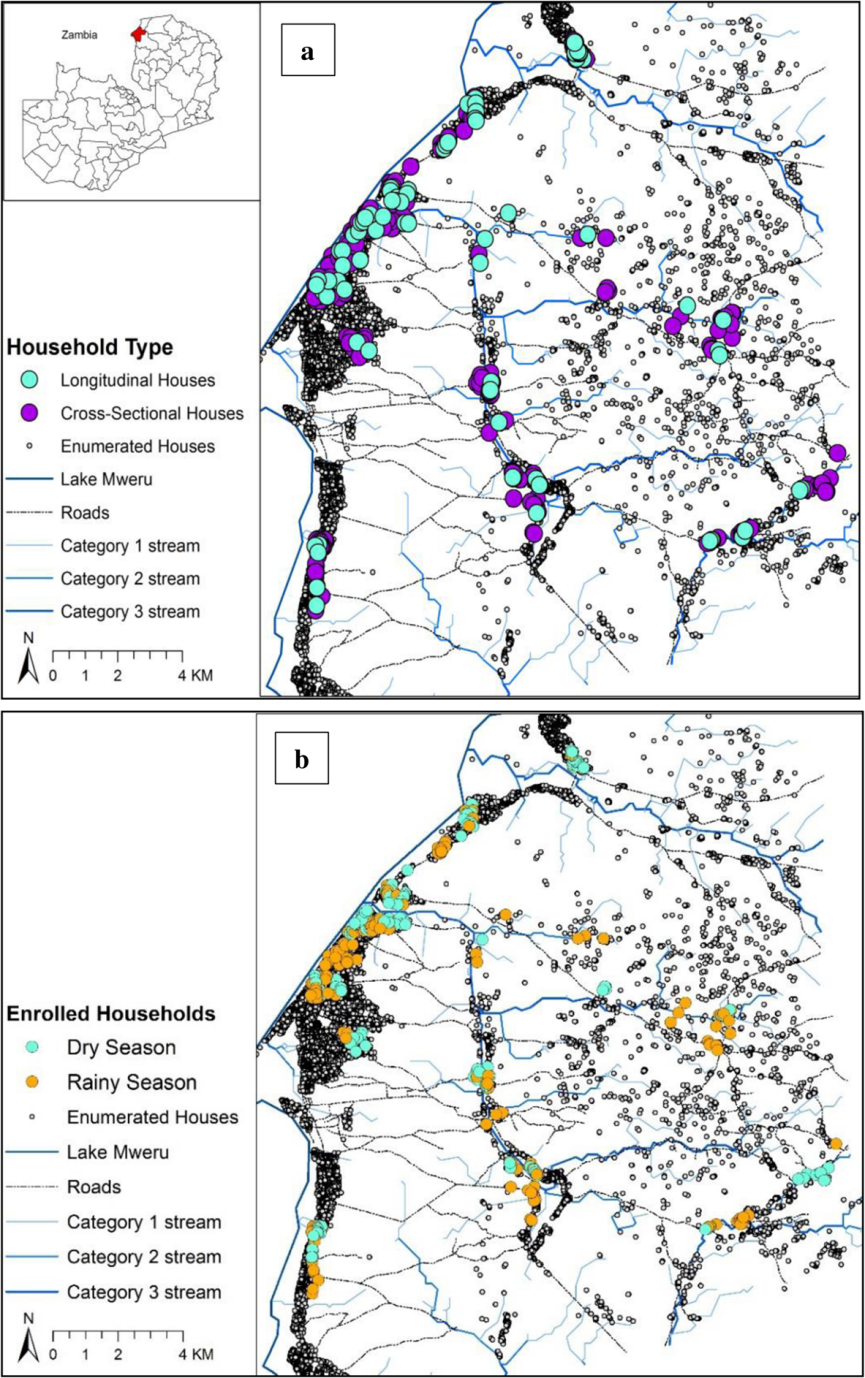
Maps of location of Nchelenge in Luapula Province, Zambia and enrolled households for mosquito collections in Nchelenge, by study and season. Households were visited between April 2012 and September 2014. Longitudinal households were enrolled in April and visited every other month. Cross-sectional households were visited once in intervening months. Grey dots represent households enumerated by satellite imagery, but not enrolled. **a** Longitudinal and cross-sectional enrolled households. **b** Households enrolled by season

Nchelenge district, Luapula Province, is one area where such gains have not been evident. The district lies in the far north of the country on Lake Mweru where it shares a border with the Democratic Republic of Congo (Fig. 1). Here, malaria prevalence across all ages increased from 38 to 53 % in the 6 years between 2006 and 2012, despite increased coverage of LLINs and indoor residual spraying (IRS) [4, 7]. Malaria Indicator Survey (MIS) data from 2012 [4] and 2015 [6] identified this province as having the highest burden of malaria in children under 5 years of age in the country, with 32.5 % positive for malaria by microscopy. Recent data from community surveys between 2012 and 2014 in Nchelenge have shown prevalences as high as 48 % by RDT across all age groups and risk maps indicate widespread risk of transmission throughout the year [8].

Between 2006 and 2011, almost half a million LLINs were distributed in Nchelenge district [7] and mass distribution campaigns were carried out in 2012 and 2014 providing another 178,000 LLINs in addition to those routinely given at antenatal clinics (NMCC pers. comm*.*). Annual IRS operations began in 2006 with pyrethroids and in 2011 coverage of households targeted for spraying reached more than 90 %. However activities in 2012 and 2013 were not comprehensive due to funding shortfalls [7]. Despite these vector control activities, limited data exist on the vector bionomics of this area and, as in many areas, entomological data were not used to guide where IRS was targeted within the district.

The first published entomological data for the district come from a small number of specimens in 2011 using pyrethrum spray catches and house aspirations, showing dominance of *Anopheles funestus* (*s.l.*) with *An. gambiae* (*s.l.*) comprising less than 20 % of the vector catch [7]. In the same study, resistance to deltamethrin was reported in *An. funestus* (*s.l.*). Later studies of mosquitoes from light traps and by pyrethrum spray catch again indicated a dominance of *An. funestus* (*s.s.*) (83 %) with smaller catches of *An. gambiae* (*s.s.*) and *An. leesoni* [9]. Studies of mitochondrial DNA demonstrated presence of both *An. funestus* Clade I and II in the area with the more common widespread Clade I comprising 80 % of collections. Clade II had, until this point, only been found in Mozambique and Madagascar [10, 11]. Detailed insecticide resistance studies demonstrated high levels of resistance to both pyrethroids and carbamates, with susceptibility to organophosphates and DDT in both *An. funestus* clades. Intensity assays indicated greater levels of resistance to deltamethrin than to bendiocarb [12] and synergist assays revealed increased expression of oxidases as the probable mechanism of resistance in the population. Assays conducted in April 2015 on F-1 progeny of *An. gambiae* (*s.s.*) showed a very high frequency of survival on deltamethrin (mortality 2.8 %, *n* = 71), low levels of resistance to bendiocarb (mortality 94.6 %, *n* = 56) and almost full susceptibility to the organophosphate pirimiphos-methyl (mortality 98 %, *n* = 100) (Coetzee, unpublished data).

During the dry season of 2012 and wet seasons of 2012 and 2013, short-term intensive collections were made in spatially distinct villages in Nchelenge by Das et al. [13] and confirmed species compositions: *An. funestus* (*s.s.*) dominated indoor collections, with *An. gambiae* (*s.s*.) an apparent secondary vector across the district. However, spatial heterogeneity was evident with catches from inland villages harbouring higher vector counts than those villages along the lake shore. Infection rates and estimated annual entomological inoculation rates (EIRs) were higher in *An. funestus* (*s.s*.) than *An. gambiae* (*s.s*.) and were shown to vary by season and year. High anthropophagy was also evident in both species [13].

Results from these earlier small-scale studies partially explain the high burden of malaria in Nchelenge district and the widespread transmission [8]. However, more comprehensive studies are necessary to fully understand the relationship between vector bionomics, household level risk factors and vector control. Studies by Das et al. [13] were limited to a few weeks in the dry and wet seasons of 2012 and 2013 and to selected villages either at the lakeside or inland. No risk analyses were carried out. Detailed spatio-temporal analyses of vector densities across the district and throughout the year may highlight key foci of higher transmission, which could be targeted for control [14, 15] and guide the appropriate timing for roll out of vector control interventions [16–18]. The current study, carried out as part of the Southern Africa International Centers of Excellence in Malaria Research project [19], therefore aimed to fill these knowledge gaps by describing the vector bionomics across Nchelenge district from monthly household collections over a two and a half year period, collecting household characteristics to identify measures associated with protection of individuals against bites and determining the effectiveness of current tools [20–25]. These will provide insight into malaria transmission dynamics in the district and help guide deployment of appropriate interventions.

## Methods

### Study site

The study was conducted in Nchelenge district, Luapula Province in northern Zambia (Fig. 1), with the main town located on the lakeside (9°19.027'S, 28°44.303'E), at an elevation of 954 m above sea level. The district borders Lake Mweru on the western side; the border between the Democratic Republic of Congo and Zambia cuts through the lake. The shoreline has extensive swamps and the inland area has a network of streams that lead to the lake. Nchelenge experiences a warm rainy season from November to April/May, followed by a cool dry season from May/June to August and a hot dry season from September to October/November. The main activity in the area is fishing between April and November on the lakeside during the dry season and farming inland between December and March during the rains and when an annual fishing ban is imposed. The main crops grown are maize and cassava. Total monthly rainfall varies from 0 mm in the dry season to 210 mm in the rainy season. Average monthly temperatures do not vary greatly through the year, averaging about 24 °C but daily fluctuations can be large with lows of 11 °C in the cold season in July and highs of 34 °C in October.

### House selection

Collections of mosquitoes were conducted in households selected for the ICEMR project studies as described elsewhere [8]. In brief, a 1 × 1 km grid was superimposed on satellite imagery of the district and households were geolocated on the imagery. Households were then randomly selected from grids across the district for either a longitudinal (cohort) study, visited every other month, or for repeated cross-sectional surveys, visited once in the intervening months through both wet and dry seasons (Fig. 1).

**Fig. 2:**
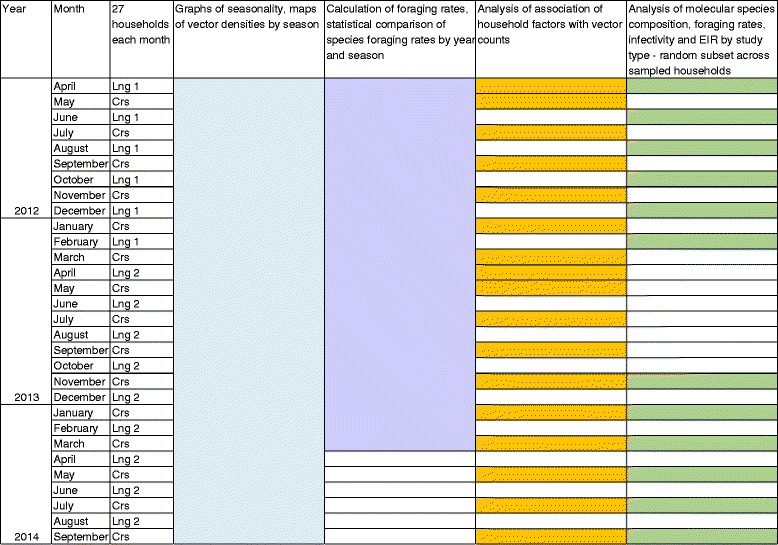
Scheme of study set-up and use of specimens for analyses. *Abbreviations*: Lng1, first longitudinal study; Lng2, second longitudinal study; Crs, cross-sectional study

Selected households were visited once per month, from April 2012 to September 2014. A first cohort of 27 longitudinal households were sampled every other month from April 2012 to February 2013 and a second cohort of 27 longitudinal households was sampled every other month from April 2013 to August 2013. On intervening months from May 2012 to September 2014, 27 different households were sampled under the cross-sectional study each month (Fig. 2). These monthly data spanning two-and-a-half years were combined to describe seasonality throughout the year, and to create district maps of vector densities by season. As the study spanned 30 months, comparisons of foraging rates of different vectors by season and location within the district were restricted to a 24 month period representing two wet and two dry seasons. To investigate the association of household factors and presence of vector control with vector counts, data were restricted to the first household visits, i.e., all those under the cross-sectional study and the first visit to the longitudinal households. Repeated visits by a health care team may result in changes in personal protection behaviour by occupants [26], and so follow-up visits as part of the longitudinal study were omitted from the models. Determination of species composition molecularly, human blood indices, annual foraging rates, *Plasmodium* infectivity and therefore annual EIR by molecularly-confirmed species was restricted to collections conducted for 1 year under the first longitudinal study (April 2012 to February 2013) and for 1 year of cross-sectional studies (November 2013 to September 2014), thus representing collections from one wet and one dry season, for 2 years. Figure 2 depicts the study set up and use of samples to address each aspect.

### Mosquito collections and collection of household data

Mosquito collections were carried out using CDC miniature light traps (The John W. Hock Company, Gainesville, Florida, USA). In the main sleeping structure of each household, light traps were set 1.5 m high at the foot of an occupied bed net. Traps were switched on at 18:00 and tied and switched off at 06:00 the following morning. At the time of trap collection, consenting household heads or their representative were interviewed to collect information on household characteristics. Data were collected on household construction, socio-economic factors, number and age of household residents, presence and use of personal protection measures against mosquitoes and recent indoor residual spray activities. Data were entered on tablets using Open Data Kit data capture software (ODK, www.opendatakit.org) and paper forms. Study data were uploaded into and managed using REDCap (Research Electronic Data Capture) software hosted at Johns Hopkins Bloomberg School of Public Health [27].

Rainfall and temperature data were collected from a HOBO Micro Station (Onset Computer Corporation, Bourne, MA) set within the study area. These data were used to determine start and end of rainy seasons for subsequent seasonal analyses.

### Mosquito processing

Traps were processed within the field house in Nchelenge where specimens were killed by freezing, identified morphologically to mosquito genus and sex and enumerated. Samples were stored dry on silica and taken once per month to laboratories at the Tropical Disease Research Centre in Ndola for further processing where morphological identifications of all anopheline mosquito species from both longitudinal and cross-sectional households were completed using standard keys [28, 29].

### Molecular identification of mosquitoes

Due to the large numbers of anophelines caught, a subset of the collections underwent molecular confirmation of identities. Total catches per household and month were calculated. From the collections of the longitudinal study carried out between April 2012 and February 2013 (which spanned both the wet and dry seasons), a maximum of 50 specimens per collection were selected to undergo molecular analysis. Additionally, from 6 months of collections from cross-sectional study households between November 2013 and September 2014, a maximum of 25 specimens of morphologically identified *An. funestus* (*s.l.*) and all *An. gambiae* (*s.l.*) per trap were processed for molecular identification (Fig. 2). DNA from mosquito abdomens was extracted using a modified salt extraction method [30], then PCR was used for identification of *An. funestus* or *An. gambiae* sibling species [31, 32] and amplicons visualised on 2.5 % agarose gels. Those specimens for which no amplification product was observed following either the *An. funestus* or *An. gambiae* PCR, were then assessed with a PCR targeting the anopheline ribosomal DNA intergenic spacer 2 (ITS2) [32, 33], modified by Das et al. [13]. To verify a subset of sample identities, the *An. funestus* or *An. gambiae* diagnostic amplicons were sequenced using the respective universal, *An. gambiae* (*s.s.*) or *An. funestus* (*s.s.*) PCR primers.

### Blood meal analysis

All samples that underwent molecular species identification, were also analysed by PCR for presence and identification of host blood using a multiplex PCR targeting the cytochrome *b* region of mitochondrial DNA and also a more sensitive PCR and restriction fragment length polymorphism (RFLP) assay [34–36].

### Detection of sporozoite positive mosquitoes

Infectivity rates were calculated from the same subsample of molecularly identified specimens collected for the longitudinal collections between April 2012 and February 2013. The heads and thoraces of all female mosquitoes from this subsample were sent to laboratories at Macha Research Trust, Southern Province, Zambia. These underwent ELISA to detect *P. falciparum* circumsporozoite antigens [37] using antibodies and an adapted protocol from MR4 (MRA-156, MR4, ATCC® Manassas Virginia, USA). Homogenates of samples that were shown to be positive, were boiled for 10 minutes and re-analysed using the same ELISA to denature any cross-reacting antigens which may result in false positives [38]. The samples selected for species identifications under the cross-sectional study underwent PCR to detect *P. falciparum* from abdominal DNA extractions [39].

### Calculation of human biting rates, human blood indices and entomological inoculation rates

Previous studies have shown correlation of human biting rates (the mean number of bites per person per night) calculated from light traps and human landing catches (HLCs) [40–43]; however, there has been much debate as to how close this correlation may be [40, 44–49]. In some areas HLCs were found to catch more vectors indoors [46] whilst others reported light traps to be more sensitive [40, 50]. In light of this uncertainty, catches per light trap were not adjusted and foraging rates were calculated as the mean number of *An. funestus* (*s.s.*) or *An. gambiae* (*s.s.*) caught per trap/night, without adjusting for room occupancy [51]. These rates were calculated over the 24 month sampling period between May 2012 and April 2014, covering two dry and wet seasons and combining both longitudinal and cross-sectional collections. These data were used to compare Williams mean biting rates between vectors by season and by year (Fig. 2). Foraging rates were also calculated separately for one year of the first longitudinal study, between April 2012 and February 2013 to calculate EIRs (see below).

For those mosquitoes identified molecularly under both studies, the human blood index, or proportion of fed mosquitoes that had human blood, was calculated by species.

The proportion of mosquitoes found to be sporozoite positive by standard ELISA methods [37] from the collections made for the period between April 2012 and February 2013 under the longitudinal study was calculated, and multiplied by the foraging rate per annum from those same households to estimate combined EIRs for both vectors and separately for each species. The boiling of the ELISA homogenate was expected to remove false positives that have been shown to arise following the standard CSP ELISA protocol [38]. Infection rates were therefore determined by heated ELISA as opposed to PCR which was carried out on the whole mosquito and so were not specific to the infectious sporozoite stage of *P. falciparum*.

### Spatial mapping of vectors

All households in the study area were digitized based on high-resolution satellite imagery from 2011. When a household was enrolled in the study, the GPS coordinates of that household were recorded using a handheld Android tablet and the collections subsequently geolocated. A Digital Elevation Model (DEM) based on the Shuttle Radar Topography Mission (SRTM) version 3 was processed in ERDAS Imagine 2011 software (ERDAS Inc, Bethesda, MD, USA) and imported into ArcGIS version 10,2 (ESRI, ArcGIS, Redlands, California, USA). The ArcHydro Tools module was used to categorize streams based on the Strahler classification system; category 1 streams are the smallest and category 10 the largest, although in the study area the largest streams were category 4. The roads and boundary of Lake Mweru were digitized from the high-resolution satellite imagery. Households that were enrolled in the study and had mosquito collections were mapped, allowing for vector abundance and species composition to be depicted each month. Maps were created using ArcGIS version 10.2 (ESRI, ArcGIS, Redlands, CA, USA).

### Statistical analyses

Numbers of female *An. funestus* (*s.l.*) and *An. gambiae* (*s.l.*) per trap/night were log-transformed [ln(n + 1)] to account for the non-normal distribution of data and the high occurrence of zeroes, and Williams means (Mw) of each species per month [52, 53] were calculated. These values were used for displaying seasonality. Mw monthly catches were compared between the two vectors, by season, using Wilcoxon signed rank sum tests. Mw of each species by season and by location categorised as ‘lake-side’ or ‘inland’, designated to be more than 3 km from the lake, were also calculated. Statistical analyses were carried out in STATA 13.1 (StataCorp. 2013. Stata Statistical Software: Release 13. College Station, TX: StataCorp LP, USA).

The entomological outcomes were merged with household survey data to identify household characteristics associated with increased counts of both *An. funestus* and *An. gambiae* mosquitoes. Zero inflated negative binomial regression models were implemented for each anopheline species. These regressions are mixture models, with a binary component to account for the excess abundance of zero counts (mosquito counts = 0 *vs* > 0) and a negative binomial component to model the full range of counts. The negative binomial model is used for the counts as an alternative to Poisson to further account for over-dispersion. Negative binomial regression results are presented as incidence rate ratios (IRR) after controlling for the excess (inflated) zero count *versus* greater than zero count behaviour. Backward variable selection was implemented and a full model was created retaining all significant main and interaction effects at the *P* ≤ 0.05 level. Statistically significant effects at the *P* ≤ 0.10 level from univariate models were considered in the backward selection process. Results of univariate models also are presented. Household variables examined were number of household residents, number of LLINs per house, percent of residents protected by LLINs, percent of households that received IRS in the last 12 months, number of households that burned a fire the night before, presence of open or closed eaves, metal or thatch roof, use of open well as main source for water, proximity to Lake Mweru (lakeside vs inland) and the season of the visit.

Models are presented/interpreted independently by species.

## Results

### Species composition

A total of 9,090 anophelines and 2,614 culicines were caught from indoor light traps under both longitudinal and cross-sectional studies carried out between April 2012 and September 2014 in Nchelenge, from a total of 770 traps set across 479 different households (see Fig. 1 for map of sampled households). Of the 9,090 anophelines, 8,929 were female and household collections ranged from 0 to 230 female anophelines per trap night. Of these, 8,148 female *An. funestus* (*s.l.*) (89.6 % of the total catch) and 749 female (8.2 %) *An. gambiae* (*s.l.*) were identified morphologically. Thirty *An. coustani*, and one specimen each of *An. maculipalpis* and *An. pharoensis* were also collected during the study.

A total of 1,868 female anophelines were caught from the longitudinal households from the 6 months’ worth of collections between April 2012 and February 2013. A subsample of 852 (45.6 %) underwent species identification by PCR: 771 specimens (90.5 %) were *An. funestus* (*s.s.*), 74 (8.7 %) *An. gambiae* (*s.s.*), 6 *An. leesoni* and 1 *An. longipalpis*.

Of the 1,332 female anophelines caught between November 2013 and September 2014 from cross-sectional houses, 713 (53.5 %) specimens were selected for analysis by PCR to confirm species identities. Of these, however, DNA was successfully extracted from only 517 specimens, likely due to storage conditions of the specimens and subsequent DNA degradation. As the specimens that were successfully analysed were no longer equally representative across month and location of collection, species composition could not be determined using these samples and so molecular assays were only used to confirm morphological identifications. From these samples, 400 of 406 morphologically identified as *An. funestus* group were confirmed as *An. funestus* (*s.s.*) (98.5 %)*,* with the remainder being *An. gambiae* (*s.s.*). Of 111 specimens morphologically identified as *An. gambiae* complex, 110 (99.1 %) were shown to be *An. gambiae* (*s.s.*) by PCR, with the remaining specimen being *An. funestus* (*s.s.*)*.* Sequencing of a subset of specimens confirmed the identities of molecularly identified *An. funestus* (*s.s.*) and *An. gambiae* (*s.s.*). The molecular assays of specimens from both the longitudinal and cross-sectional studies, indicated that 97.4 and 92.2 % of morphologically identified specimens of *An. funestus* (*s.l.*) and *An. gambiae* (*s.l.*), respectively, were correctly identified. Unless stated otherwise, therefore, results henceforth refer to morphological identifications.

### Foraging rates and seasonality

**Fig. 3:**
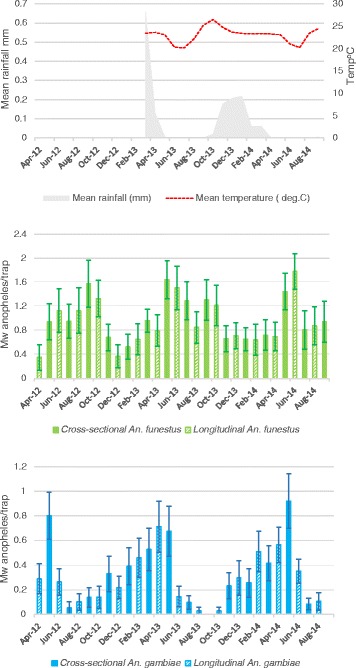
Seasonality of *An. funestus* (*s.l.*) and *An. gambiae* (*s.l.*) in Nchelenge District, May 2012 to April 2014. Note, climate data collection within the study site only commenced in 2013. Means are monthly Williams mean (Mw) of counts per trap. Solid bars refer to cross-sectional collections; hatched bars refer to longitudinal households. Longitudinal households comprised two sets of households, the first set was enrolled in April 2012 and the second in April 2013

For the two year period between May 2012 and April 2014, a total of 6,848 vectors from 617 traps were caught across both longitudinal and cross-sectional study households to give a mean nightly catch of 11.10 (95 % CI: 8.82–13.37) vectors per house. The arithmetic mean foraging rate for *An. funestus* was calculated to be 10.06 per night (95 % CI: 7.85–12.27), whilst that for *An. gambiae* was 1.04 (95 % CI: 0.77–1.31). However, sets of data for both species were highly skewed with 303 of 479 households (63.3 %) never having *An. funestus* and 390 (78.5 %) having no catches of *An. gambiae.* Nine households had more than 150 *An. funestus* in a trap with the highest catch being 226 in a single night during this two year period. In contrast, catches of *An. gambiae* did not exceed 32 on any given trap night. Williams means of catches of the two species for each season and by month were calculated (Fig. 3, Table 1). Catches of *An. funestus* exceeded those of *An. gambiae* across all seasons both in 2012 and 2013 (Wilcoxon signed rank test; 2012 dry: *Z* = 5.10, *P* < 0.001; 2012 wet: *Z* = 4.23, *P* < 0.001; 2013 dry: *Z* = 7.31, *P* < 0.001; 2013 wet: *Z* = 4.62, *P* < 0.001).

**Table 1:** Williams mean (Mw) catch (95 % CI) and maximum number caught in a single trap of *An. funestus* (*s.l.*) and *An. gambiae* (*s.l.*) by season. Collections were made from CDC miniature light traps from May 2012 to April 2014 in Nchelenge district, Zambia, and presented by season (Dry: May to October, Rainy: November to April). Catches are summarized from all longitudinal and cross-sectional study households

		*An. funestus* (*s.l.*)		*An. gambiae* (*s.l.*)	
		Mw (95 % CI)	Maximum catch (*n*)	Mw (95 % CI)	Maximum catch (*n*)
2012	Dry	1.14 (0.84–1.44)	226	0.28 (0.17–0.40)	17
	Wet	0.75 (0.58–0.93)	139	0.39 (0.28–0.51)	32
2013	Dry	1.32 (1.04–1.60)	96	0.19 (0.09–0.29)	32
	Wet	0.76 (0.57–0.94)	110	0.33 (0.23–0.42)	17

All Mw catches of *An. gambiae* (*s.l.*) were significantly lower than those of *An. funestus* (*s.l.*) within each season

From the 1 year period of the longitudinal households, the 1,868 female anophelines morphologically identified as *An. funestus* or *An. gambiae* were collected from 148 trap-nights, to give an arithmetic mean foraging rate of 12.62 mosquitoes per night (95 % CI: 6.13–19.11).

### Blood-fed rates and HBI

Abdominal status as fed or not fed was visually scored for 862 samples from the longitudinal study. Fifty-seven (6.6 %) were visually assessed to contain blood.

From the cross-sectional study, blood meal analysis by PCR was successfully carried out on 517 molecularly identified samples of the 1,332 anophelines caught in cross-sectional houses between November 2013 and September 2014. Using both the Kent and Norris [35] and Fornadel and Norris [36] PCR assays, a total of 30 (5.8 %) had sufficient host DNA to confirm that they had all fed on humans, to give an HBI for both *An. funestus* and *An. gambiae* of 1.0.

### Infectivity rates and entomological inoculation rates

Of the 852 molecularly identified specimens from the first longitudinal study, 739 female anophelines were analysed for sporozoite infections by ELISA, subsampling by month and household. The remaining specimens were missing head and thoraces. Of the 739 specimens analysed, 670 were identified molecularly as *An. funestus* (*s.s*.) and 69 as *An. gambiae* (*s.s*.). Thirteen specimens were found to be CSP positive by the standard CSP ELISA and all 13 remained positive following heating of the homogenate, giving an overall infection rate of 1.8 %, of which eleven were *An. funestus* (*s.s*.) and two *An. gambiae* (*s.s*.), to give infection rates of 1.64 and 2.90 % for the two species, respectively.

From the cross-sectional study households, those specimens for which a molecular species identity was determined underwent *P. falciparum* PCR. Seven of 517 specimens analysed (1.35 %) were positive for *P. falciparum*. Infection rates were 1.0 % (4 specimens out of 401) and 2.6 % (3 of 116) in confirmed *An. funestus* (*s.s*.) and *An. gambiae* (*s.s*.), respectively.

Using the infection rate by ELISA from one year of the longitudinal study in 2012–2013 and the foraging rate from those households, the entomological inoculation rate for Nchelenge was calculated as 81.04 infectious bites per person per annum (ib/person/pa). EIRs for *An. funestus* (*s.s*.) and *An. gambiae* (*s.s*.) were calculated to be 71.71 ib/person/pa and 6.93 ib/person/pa, respectively.

### Spatial distribution of vector species

**Fig. 4:**
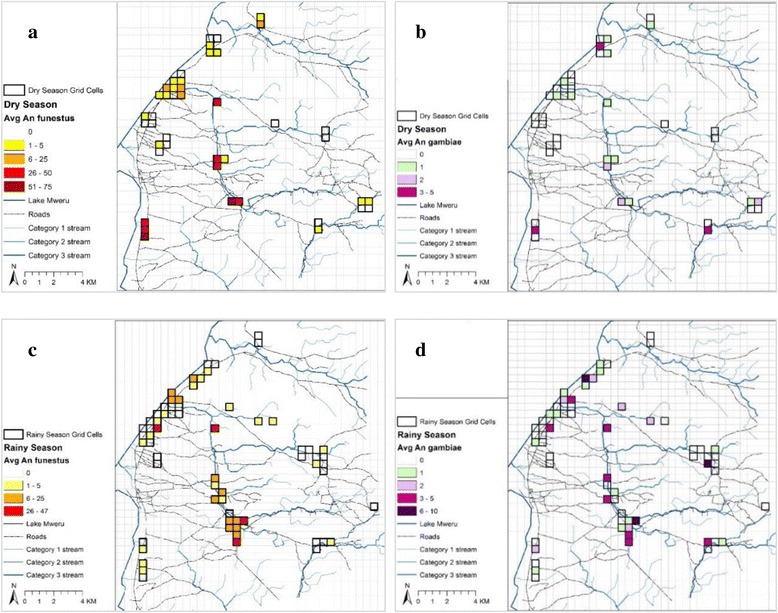
Map of average household *An. funestus* and *An. gambiae* counts aggregated to 500 m grid cells in Nchelenge per season, April 2012- September 2014. Maps here reflect catches from all cross-sectional visits and the first visit of the longitudinal studies. **a**, **b** Dry season abundance. **c**, **d** Wet season abundance

The location of enrolled cross-sectional and longitudinal households in which traps were set in the rainy and dry season are depicted in Fig. 1, against the total distribution of enumerated household structures in Nchelenge. The spatial changes in vector densities by season are depicted in Fig. 4. Grid cells 500 × 500 m were overlaid on the study area and mosquito counts aggregated to the grid cell in order to visualize spatial variation in mosquito counts each season. The maps highlight the distribution of *An. funestus* with the highest cell counts (maximum average of 75 mosquitoes) in the dry season inland from the Lake (Fig. 4a, c). *Anopheles gambiae* counts were lower overall and not as ubiquitous as *An. funestus*; means rarely reached above 6 mosquitoes per trap. Higher average densities were evident in the rainy season (maximum average of 10 mosquitoes) (Fig. 4b, d).

Williams means of catches of *An. funestus* were greater inland than near the lake during both the dry (inland Mw 1.89, 95 % CI: 1.35–2.42; lakeside Mw 1.04, 95 % CI: 0.76–1.31) and rainy season (inland Mw 1.11, 95 % CI: 0.8–1.43; lakeside Mw 0.37, 95 % CI: 0.21–0.53). *Anopheles gambiae* catches, however, were not significantly different between the lakeside and inland areas during either season (dry season: inland Mw 0.39, 95 % CI: 0.19–0.59; lakeside Mw 0.18, 95 % CI: 0.07–0.29; wet season: inland Mw 0.39, 95 % CI: 0.19–0.59; lakeside Mw 0.18, 95 % CI: 0.07–0.29) (Additional file 1: Table S1).

### Household level risk analysis

Household level factors associated with indoor densities of *An. funestus* (*s.l.*) and *An. gambiae* (*s.l.*) were analysed separately and restricted to the cross-sectional study and the first visit of the longitudinal study. Households had an average of 5.5 residents, and average of 1.2 bednets per house, with an average of 2.86 persons reportedly sleeping under an LLIN the night before (Table 2). Only 35 % of households reported receiving IRS in the past 12 months. About half (47 %) were visited in the rainy season, and 30 % reported an open well as their main water source (Table 2). Results of the zero inflated negative binomial regression for *An. funestus* and *An. gambiae* are presented in Tables 3 and 4. After accounting for excess zeroes, the negative binomial component modelling mosquito counts indicated that *An. funestus* counts were 60 % lower in the rainy season than the dry season (IRR = 0.405; 95 % CI: 0.198–0.825), after controlling for IRS and proximity to Lake Mweru (Table 3). The univariate model results for *An. funestus* did not reveal any other significant factors other than a slightly more pronounced rainy season effect; positive counts 65 % lower in the rainy season (IRR = 0.358; 95 % CI: 0.204–0.631).

**Table 2:** Household characteristics of enrolled households, Nchelenge Zambia 2012–2014 for mosquito collections. Data represent all cross-sectional study households and first visit to longitudinal households

	*n* (%)	Missing (*n*)
# Houses	351	
Mean no. of people per house (min–max)	5.52 (2–13)	101
Mean no. of nets per house (min–max)	1.21 (0–8)	101
Mean no. of persons under LLINs (min–max)	2.86 (0–10)	101
% People protected by nets	52	101
% Received IRS, last 12 months	35	8
% Fire night before	13	98
% Open eaves	97	98
% Metal roof	4	98
Open well	30	
Household visited in rainy season (%)	47	
Within 3 km of Lake Mweru (%)	68

**Table 3:** Household factors associated with *An. funestus* counts in zero inflated negative binomial model, Nchelenge, 2012–2014. Univariate and multivariable models include data from cross-sectional and first visit to longitudinal study households. Presented are results for the negative binomial positive counts (counts > 0) as incidence rate ratios (IRR)

Negative binomial counts > 0	Univariate			Multivariable		
Variable	IRR	95 % CI		IRR	95 % CI	
Persons sleeping in the house	1.100	0.910	1.320	–	–	–
ITNs owned per house	1.111	0.680	1.814	–	–	–
IRS, ever	1.057	0.557	2.006	1.073	0.495	2.325
Closed eaves	4.734	0.378	59.215	–	–	–
Metal roof	0.164	0.019	1.436	–	–	–
Fire burned previous night	0.675	0.220	2.073	–	–	–
Rainy season	0.358*	0.204*	0.631*	0.405*	0.198*	0.825*
Open well	0.811	0.449	1.467	–	–	–
Within 3 km of Lake Mweru	1.013	0.553	1.857	0.813	0.367	1.801
Interaction: season and lake distance	0.724	0.209	2.506	–	–	–

*Outcomes indicated with an asterisk are statistically significant

**Table 4:** Household factors associated with *An. gambiae* counts in zero inflated negative binomial model, Nchelenge, 2012–2014. Univariate and multivariable models includes data from cross-sectional and first visit to longitudinal study households. Presented are results for the negative binomial positive counts (counts >0) as incidence rate ratios (IRR)

Negative binomial counts > 0	Univariate			Multivariable		
Variable	IRR	95 % CI		IRR	95 % CI	
No. of persons sleeping in house	0.902	0.740	1.100	–	–	–
No. of ITNs per house	0.559*	0.349*	0.897*	–	–	–
IRS, ever	0.902	0.440	1.851	1.039	0.444	2.433
Closed eaves	0.217	0.016	2.956	0.102*	0.011*	0.904*
Metal roof	3.197	0.287	35.610	–	–	–
Fire burned previous night	0.810	0.252	2.608	–	–	–
Open well	2.229*	1.078*	4.608*	2.257*	1.068*	4.766*
Rainy season	1.420	0.714	2.824	1.000	0.360	2.776
Within 3 km of Lake Mweru	0.601	0.291	1.243	0.204*	0.072*	0.581*
Interaction: Season by inland/lakeside	0.634	0.145	2.766	3.932*	1.024^a^	15.096*

*Outcomes indicated with an asterisk are statistically significant

A second zero inflated negative binomial model was conducted for *An. gambiae*. Univariate analysis indicated that for the negative binomial component modelling mosquito counts, *An. gambiae* counts were 44 % lower in households with a higher number of LLINs in a household (IRR = 0.559; 95 % CI: 0.349–0.897) and *An. gambiae* counts were over twice as high if a house used an open well for its water source (IRR = 2.229; 95 % CI: 1.078–4.608), after accounting for excess zeroes. In the full model, number of nets were no longer significantly associated with lower *An. gambiae* catches, but use of open wells remained significant (IRR = 2.257; 95 % CI: 1.068–4.766). Additionally having closed eaves was associated with 90 % lower *An. gambiae* counts (IRR = 0.102; 95 % CI: 0.011–0.904), and 80 % lower counts were in households located within 3 km of Lake Mweru (lake side) (IRR = 0.204; 95 % CI: 0.072–0.581). There was also a statistically significant interaction between season and being located closer to Lake Mweru; in the rainy season, there were higher *An. gambiae* counts in houses located within 3 km of Lake Mweru (lake side) (IRR = 3.932; 95 % CI: 1.024–15.096) (Table 4).

## Discussion

The results from this study confirm previous findings that *An. funestus* (*s.s*.) dominates in Nchelenge district with *An. gambiae* (*s.s*.) playing a more minor role in transmission of malaria [13]. *Anopheles funestus* is the dominant malaria vector in much of southern Africa, but its spatial distribution is wide-reaching across many parts of Sub-Saharan Africa [54]. Recent studies indicate this species may be increasing in densities and dominating parts of East Africa [55, 56], likely due to increasing levels of insecticide resistance and studies from Nchelenge would support this. Both *An. funestus* (*s.s*.) and *An. gambiae* (*s.s*.) show high levels of endophagy and anthropophagy [28, 29] and in combination can result in high levels of transmission [57]. This is reflected in the entomological inoculation rates for the two species, 71.71 ib/person/pa and 6.93 ib/person/pa for *An. funestus* (*s.s*.) and *An. gambiae* (*s.s*.), respectively. These EIRs are considered moderately high compared to estimates from other parts of Africa [49], where some countries such as Senegal have in the past recorded EIRs of several hundred [58]. The results reported here are considered conservative as only a spatially representative subset of samples were analysed by ELISA for circumsporozoite proteins, and a larger sample may have revealed higher infection rates. Temporally and spatially focal collections in Nchelenge have previously revealed infection rates as high as 3.9 % [13] and 9.2 % [12]. The low infection rates reported in the current study across the district could also be attributed to low daily temperatures during the winter months. At the end of June and beginning of July mean monthly temperatures average about 20 °C; between midnight and sunrise temperatures do not exceed 15 °C. In July 2014 the lowest daily external temperature recorded was 10.5 °C. Although it is likely that mosquitoes were able to find warmer microclimates such as within houses, it can be envisaged that parasite development during these times of the year are extended and fewer mosquitoes survive the extrinsic incubation rate of *P. falciparum*, resulting in lower infection rates. Estimates of EIR in Zambia are not widely reported, but those calculated in this paper are equivalent to that previously documented in the area [13]. The 13 *P. falciparum* positive samples collected from the current study were only collected in 3 months of the year (June, October and January) and six of these samples were collected equally from two households on the same date. This would suggest that occurrence of infectious mosquitoes is spatially heterogeneous and seasonal and may explain why earlier studies reported higher infection rates from more focal and temporally distinct collections in the district. Clustering of malaria infections and hotspots or foci of transmission in Africa are widely acknowledged, [14, 15, 59–63], and so it follows that presence of malaria-positive vectors would also be focal. Not surprisingly, the coincidence of infections in people and in mosquitoes has been demonstrated for other mosquito borne diseases [64]. The EIRs reported here exceed those estimated at a wider spatial level, by the Malaria Atlas Project for any area of Zambia [65]. This may explain why Nchelenge has some of the highest malaria prevalences of the country [8].

Spatio-temporal trends of the two species were shown to vary. During the rains, catches of *An. funestus* (*s.s*.) were significantly lower than in the dry season across the study area. *Anopheles gambiae* (*s.s*.) populations, however, increased with onset of the rains but only significantly so at the lakeside. Although this study did not include larval abundance of the two species, these seasonal and spatial trends are likely due to variation in breeding site preference of the two species; *An. funestus* larvae tend to inhabit larger, more permanent water bodies that flood and are washed out during the rains, whilst *An. gambiae* thrive in open water bodies that may be formed by pooling of water following rainfall [66]. This pooling is likely to occur close to the lake, and from expansion of water bodies next to open wells, commonly used in this area. During the dry season, when the typical breeding sites for *An. gambiae* (*s.s*.) dry up, *An. funestus* (*s.s*.) thrive, presumably as their breeding sites stabilise and expand with decreased water flow and perhaps increased vegetated harbourage. Mean household catches of *An. funestus,* however, predominated over *An. gambiae* (*s.s*.) during both wet and dry seasons. Although ecological niche separation has been reported for these two species in Kenya [67], in Nchelenge a number of households had catches of both species within the same collection, suggesting that breeding sites for both are within flight distances of households. The abundance of *An. funestus* through the year combined with the surge in number of *An. gambiae* during the rains in some areas, results in perennial presence of vectors in the district. Together, these two endophagic and highly anthropophagic species could contribute to greater transmission than evident by the EIR estimates of either single species alone [67].

Although the multivariable negative binomial regression model indicated no significant association of catch density of *An. funestus* with distance from the lake having accounted for other variables in the model, there were large house to house heterogeneities in the collections. Mean catches were generally higher inland and the three households with the highest catches, with more than 190 mosquitoes per trap/night, were located along the Kenani Stream that feeds into the lake. Previous studies had also indicated presence of *An. funestus* in large numbers along this stream [13]. Household densities of *An. gambiae,* having accounted for season, were shown to be lower close to the lake than inland. IRS programmes in Nchelenge have largely focused on the high human population density areas next to the lake and these inland areas have not been targeted in the past, largely due to difficulties of access and limitations of financial support [68]. Combining household densities throughout the year for both vectors, indicates that this lakeside part of the district has far fewer vectors, and presumably transmission levels, than the inland areas.

IRS campaigns generally roll out just prior to the rains with the expectation that this would target mosquito populations that are low and prevent surges in transmission. The data here show that, while such a strategy may be effective for *An. gambiae*, which surge at the lakeside once rains begin, *An. funestus* household densities are at some of their highest during this time and substantial levels of transmission are likely to already be evident. Pyrethroids, DDT and a new micro-encapsulated formulation of an organophosphate, pirimiphos-methyl, used for IRS are generally effective for about 6 months [69, 70]. In the case of Nchelenge, effectiveness of an IRS scheme deployed in October would wane in the dry season when *An. funestus* populations again thrive. In addition, the community in Nchelenge are relatively mobile and move from the lakeside fishing areas to more fertile agricultural areas inland during the fishing ban, which is at the time of the rains. This scenario may reduce the protective efficacy for those residents who move during times of farming to unsprayed inland homes where vector populations are high. Therefore, an effective IRS programme in Nchelenge would require two spray rounds per year. Another threat to the effectiveness of the control measures, is the extremely high levels of insecticide resistance in *An. funestus* and *An. gambiae* to pyrethroid and carbamate chemicals [12] that were deployed for IRS and used for impregnation of nets. Das et al. [9] reported more than 30 % of *An. funestus* being blood fed from mosquitoes caught from light traps and spray collections in households in Nchelenge. In the current study, approximately 6 % of vectors were found to be blood fed despite being caught by light traps which are designed to catch foraging mosquitoes. This would support the finding that current vector control measures have not adequately reduced mosquito exposure in Nchelenge. Data on malaria prevalence from the district have shown high transmission throughout the year, despite high bed net coverage and intermittent IRS [8].

During the study period from April 2012 to September 2014, less than 35 % of households reported being sprayed in the last year. This could be attributed to bias in responder’s recall; houses reported not to be sprayed were not verified by district-level reports in this study. This low coverage could also, in part, be explained by the fact that the study interviewed households from across the district, whilst spray teams could not feasibly access more distant inland homes. This low coverage may explain the lack of association of IRS with vector counts, even for *An. gambiae,* against which the lake-side pre-rainy season application would be assumed to be a better target. At the end of 2014, after the study concluded, the IRS campaign in the district was reinvigorated. More households along the lake and a few accessible ones inland were sprayed with a long-lasting formulation of pirimiphos-methyl to which *An. funestus* is susceptible [12], following the recommendations of the WHO to combine both LLINs and IRS to reduce malaria in areas of high endemicity [69]. As Nchelenge continues to deploy both tools, it will be necessary to evaluate the impact of these improved control campaigns. Whilst the protective efficacy of each tool against malaria in areas with insecticide-susceptible indoor foraging and resting mosquitoes has been widely shown [71, 72], the added benefit of combining LLINs and IRS in a household against malaria appears to be variable [73–79] and may depend on intervention coverage, transmission intensity, the insecticides used, level of insecticide resistance in vectors and number of rounds of IRS carried out in an area [80, 81].

Whilst approximately 85 % of homes reported having at least one net, there was a large disparity between the mean number of nets per household (1.2) and the average household occupancy (5.5). Usage was reported to be 52 %. Unlike other areas of Zambia [26], this statistic is low. The National Malaria Control Programme has mass distribution campaigns for the province in 2017. Distribution should be combined with messages within the community to encourage net use because despite the very high levels of pyrethroid resistance in the vectors, nets offer a protective barrier against mosquito bites. Other protective measures include the screening of house entry points such as eaves. Studies in the Gambia and Tanzania showed significant impact of screening houses in preventing both mosquito entry and reducing malaria cases [23–25] and in Uganda and Tanzania, reduced mosquito densities and lower malaria incidence was demonstrated in modern houses with fewer points of entry compared to traditional houses [82, 83]. In Nchelenge, screening eaves could prevent entry of *An. gambiae*. The lack of association of open eaves with *An. funestus* abundance may be due to the relatively small number of households with closed eaves, especially in less developed inland areas where *An. funestus* thrive.

Entomological and epidemiological surveillance must be maintained and used to appropriately design interventions before large control programmes are rolled out, and these indices should be used to subsequently evaluate programmes throughout the year; the seasonal changes of the two vectors demonstrated in Nchelenge, northern Zambia, in this study would indicate that the timing of vector control is crucial and that a single round of IRS may not be adequate. Furthermore, all studies in this area to date have focused on indoor foraging of mosquitoes and there are no data on whether outdoor transmission may also be a threat to the current indoor-directed vector control interventions. There is urgent need for entomological data on the extent of exophagy and exophily.

## Conclusions

This is the first study, to our knowledge, to describe the district-wide spatio-temporal characteristics of malaria vectors in Nchelenge, Luapula Province, northern Zambia. This unique long-term dataset has demonstrated the perennial co-existence of two highly anthropophagic vectors, *An. funestus* and *An. gambiae* across the district that are not currently being controlled by pyrethroid-based insecticide tools due to high levels of resistance in the vectors. Entomological inoculation rates exceeding 80 infectious bites per person per annum, make this one of the districts with the highest transmission levels in Zambia. Here, the transmission system is complex and to reduce malaria, a district-wide coordinated, site-tailored package of interventions is required.

## Additional file

Additional file 1: Table S1. Williams mean (Mw) catch (95 % C.I.) of *An. funestus* (*s.l.*) and *An. gambiae* (*s.l.*) by season and locality. Collections were made from CDC miniature light traps from May 2012 to April 2014 in Nchelenge district, Zambia and are presented by season (Dry: May to September, Rainy: November to April) and by locality (within 3 km of the lake; inland). Data represent catches from cross-sectional study households and first visit to longitudinal households. (DOCX 12 kb)

## Abbreviations

CDC: Centers for Disease Control and Prevention
CSP: Circumsporozoite protein
DDT: Dichloro-diphenyl-trichloroethane
DEM: Digital elevation model
EIR: Entomological inoculation rate
ELISA: Enzyme-linked immunosorbent assay
HBI: Human blood index
HBR: Human biting rate
HLC: Human landing catch
ICEMR: International Centers of Excellence in Malaria Research
IPTp: Intermittent preventative treatment in pregnancy
IRS: Indoor residual spray
ITN: Insecticide-treated net
LLIN: Long-lasting insecticide-treated net
MIS: Malaria indicator survey
Mw: Williams mean
NMCC: National Malaria Control Centre
ODK: Open Data Kit data management software
PCR: Polymerase chain reaction
RDT: Rapid diagnostic test
REDCap: Research electronic data capture
*s.s.*: *sensu stricto*
SRTM: Shuttle radar topography mission
WHO: World Health Organization
