# Supplementary material for: Spatio-temporal heterogeneity of malaria vectors in northern Zambia: implications for vector control
Source: Parasit Vectors. 2016 Sep 21;9:510. doi: 10.1186/s13071-016-1786-9 (PMC5031275; doi:10.1186/s13071-016-1786-9)
Supplement: Additional file 1: Table S1. — Williams mean (Mw) catch (95 % C.I.) of An. funestus (s.l.) and An. gambiae (s.l.) by season and locality. Collections were made from CDC miniature light traps from May 2012 to April 2014 in Nchelenge district, Zambia and are presented by season (Dry: May to September, Rainy: November to April) and by locality (within 3 km of the lake; inland). Data represent catches from cross-sectional study households and first visit to longitudinal households. (DOCX 12 kb) [file 13071_2016_1786_MOESM1_ESM.docx]

**Additional File 1. Table S1.** Williams mean (Mw) catch (95% C.I.) of *An. funestus* (*s.l.*) and *An. gambiae* (*s.l.*) by season and locality. Collections were made from CDC miniature light traps from May 2012 to April 2014 in Nchelenge district, Zambia and are presented by season (Dry: May to September, Rainy: November to April) and by locality (within 3 km of the lake; inland. Data represent catches from cross-sectional study households and first visit to longitudinal households.

|  | ***An. funestus* (*s.l.*)** | ***An. gambiae* (*s.l.*)** |
| --- | --- | --- |
|  | **Mean (95% CI)** | **Mean (95% CI)** |
| Rainy Season | | |
| Within 3 km of Lake Mweru | 0.37 (0.21, 0.53) | 0.27 (0.15, 0.38) |
| Inland | 1.11 (0.8, 1.43) | 0.54 (0.33, 0.74) |
| Dry Season | | |
| Within 3 km of Lake Mweru | 1.04 (0.76, 1.31) | 0.18 (0.07, 0.29) |
| Inland | 1.89 (1.35, 2.42) | 0.39 (0.19, 0.59) |
